# Population mixing for leukaemia, lymphoma and CNS tumours in teenagers and young adults in England, 1996–2005

**DOI:** 10.1186/1471-2407-14-698

**Published:** 2014-09-23

**Authors:** Marlous van Laar, Daniel P Stark, Patricia McKinney, Roger C Parslow, Sally E Kinsey, Susan V Picton, Richard G Feltbower

**Affiliations:** Division of Epidemiology and Biostatistics, School of Medicine, University of Leeds, Worsley Building, Clarendon Way, Leeds, LS2 9JT UK; St James’s Institute of Oncology, Bexley Wing, Level 04, St James’s University Hospital, Beckett Street, Leeds, LS9 7TF UK; Regional Department of Paediatric Oncology and Haematology, Leeds General Infirmary, Martin Wing, D Floor, Great George Street, Leeds, LS1 3EX UK

**Keywords:** Population mixing, Aetiology, Teenage and young adult, Adolescent, Oncology, Leukaemia, Lymphoma, Central nervous system tumours

## Abstract

**Background:**

Little aetiological epidemiological research has been undertaken for major cancers occurring in teenagers and young adults (TYA). Population mixing, as a possible proxy for infectious exposure, has been well researched for childhood malignancies. We aimed to investigate effects of population mixing in this older age group using an English national cancer dataset.

**Methods:**

Cases of leukaemia, lymphoma and central nervous system (CNS) tumours amongst 15–24 year olds in England (diagnosed 1996–2005) were included in the study. Data were obtained by ward of diagnosis and linked to 1991 census variables including population mixing (Shannon index); data on person-weighted population density and deprivation (Townsend score) were also used and considered as explanatory variables. Associations between TYA cancer incidence and census variables were investigated using negative binomial regression, and results presented as incidence rate ratios (IRR) with 95% confidence intervals (CI).

**Results:**

A total of 6251 cases of leukaemia (21%), lymphoma (49%) and CNS tumours (30%) were analysed. Higher levels of population mixing were associated with a significant decrease in the incidence of CNS tumours (IRR = 0.83, 95% CI = 0.75-0.91), accounted for by astrocytomas and ‘other CNS tumours’; however, there was no association with leukaemia or lymphoma. Incidence of CNS tumours and lymphoma was 3% lower in more deprived areas (IRR = 0.97, 95% CI = 0.96-0.99 and IRR = 0.97, 95% CI =0.96-0.98 respectively). Population density was not associated with the incidence of leukaemia, lymphoma or CNS tumours.

**Conclusions:**

Our results suggest a possible role for environmental risk factors with population correlates in the aetiology of CNS tumours amongst TYAs. Unlike studies of childhood cancer, associations between population mixing and the incidence of leukaemia and lymphoma were not observed.

## Background

A number of epidemiological studies have reported associations between childhood leukaemia and population mixing [1–4], suggesting a role for infections in disease aetiology. This association is based upon the premise that areas with high levels of population mixing are likely to exhibit higher prevalence and a greater range of infections [5]. Increased levels of population mixing can be considered as a risk factor for cancer through exposure to some unknown infectious agent(s) as proposed by Kinlen [1]. Alternatively, Greaves [4] proposes a ‘delayed infection hypothesis’ in which increased levels of population mixing provides a protective role through its development of the immune system. This hypothesis is supported by several previous studies of childhood ALL [3, 6]. The evidence of a possible infectious aetiology for childhood tumours is most established for childhood leukaemia and lymphoma [2, 7–12], but is also emerging for central nervous system (CNS) tumours [13–17] although inconsistencies in the research exist for the latter group [18–20]. Despite increasing evidence of an infectious aetiology for childhood tumours, little aetiological research has been undertaken for major cancers occurring in teenagers and young adults (TYA). Indeed, there is a paucity of epidemiological research investigating causal factors for cancer amongst TYAs [21], a population which presents with distinct forms of cancer [21, 22].

There is some evidence showing geographical variation in the incidence of TYA cancer, including an inverse association between levels of deprivation and the occurrence of leukaemia, lymphoma and central nervous system (CNS) tumours [23]. We have previously shown evidence of seasonality around the time of diagnosis for Hodgkin’s lymphoma and ‘other’ CNS tumours (subgroup 3.5 - [24]) as well as seasonality around the time of birth for those with glioma (except astrocytoma and ependymoma) [25]. In this study we investigated whether there was any evidence of an association between population mixing and these tumours occurring in 15–24 year olds using the same national dataset in England and an ecological study design. Population mixing is likely to reflect the level of circulating infections within small geographical areas, which are known to be dependent upon population density [26]. We therefore investigated the impact of adjusting for population density and deprivation on the reported association with population mixing, as both covariates were likely to be confounding factors, and possibly correlated.

## Methods

Cases of leukaemia, lymphoma and central nervous system (CNS) tumours amongst 15–24 year olds diagnosed between 1996 and 2005 in England were obtained from the national TYA database held by the North West Cancer Intelligence Service (Research Ethics Committee approval 09/H1302/37). All diagnoses were coded according to the Birch classification of cancer for TYA [24]. Age and sex-specific population figures by year and 2001 standard table census ward were obtained from the Office for National Statistics.

For each individual, the full address (including postcode) at diagnosis was geo-coded to census wards. We chose to allocate measures of population mixing, population density and deprivation to each subject based on the 1991 census. This census period overlapped with the modal exposure time for individuals included in the study and so reflected the average level of mixing attributed to the cohort diagnosed from 1996–2005, given the potential exposure time window ranged from 1971–2005. All age population mixing was measured using the Shannon Index [27] as an indicator of the diversity of incoming migrants into each ward during the year before the census, and was derived using 1991 Census Special Migration Statistics [28]. Ward level population density was person-weighted according to enumeration district to gain a more accurate measure of the average density at which most individuals live [29]. Area based deprivation levels were measured using the Townsend deprivation score calculated from levels of unemployment, household over-crowding, housing tenure and car ownership [30]. These methods are described in detail in a previous ecological analysis carried out in Yorkshire [6].

### Statistical methods

Possible associations with leukaemia, lymphoma and CNS tumour incidence and population mixing, population density, and deprivation were initially investigated using Poisson regression. Negative binomial regression was used in cases where data showed evidence of over dispersion by performing likelihood ratio tests of the scale parameter. Due to the possibility of multicollinearity occurring between population mixing, deprivation and population density, correlation between variables was assessed using Spearman’s rank coefficient. Likelihood ratio testing and Akaike’s Information Criterion (AIC) were used to determine which variables should be included in the models alongside population mixing (the base model) and whether these should be included as continuous or categorical variables [31]. We were also interested in possible threshold effects, and so additionally report categorisation of population mixing into the 1^st^, 2^nd^ to 9^th^ and 10^th^ deciles, similar to previous analyses [6, 32].

## Results

There were a total of 6251 cases of leukaemia, lymphoma and CNS tumours diagnosed amongst 15–24 year olds between 1996 and 2005 in England. Table 1 gives the number of cases and incidence rates of leukaemia, lymphoma and CNS tumours according to diagnostic subgroup.

**Table 1:** **Leukaemia, lymphoma and CNS tumours amongst teenagers and young adults in England, 1996-2005**

Diagnostic group ^a^	Cases (subgroup%)	Incidence rate (per 10 ^6^person-years)
**Leukaemia**		
*Acute lymphoid leukaemia (ALL)*	591 (45%)	9.7
*Acute myeloid leukaemia (AML)*	463 (36%)	7.6
*Chronic Myeloid leukaemia (CML)*	147 (11%)	2.4
*Other leukaemia*	98 (8%)	1.6
***All leukaemias***	**1299**	**21.3**
**Lymphoma**		
*Non-Hodgkin’s lymphoma (NHL)*	991 (32%)	16.3
*Hodgkin’s lymphoma (HL)*	2079 (68%)	34.1
***All lymphomas***	**3070**	**50.3**
**CNS tumours**		
*Astrocytoma*	629 (33%)	10.3
*Other Glioma*	195 (10%)	3.2
*Ependymoma*	99 (5%)	1.6
*Medulloblastoma*	111 (6%)	1.8
*Other Specified CNS*	702 (37%)	11.5
*Unspecified CNS*	146 (8%)	2.4
***All CNS tumours***	**1882**	**30.9**
**All leukaemia, lymphoma and CNS tumours**	**6251**	**102.5**

^a^Defined according to the Birch Teenage and Young Adult Classification Scheme [24].

*Abbreviation:* *CNS* central nervous system.

Population mixing was significantly correlated with population density (Spearman’s rank coefficient = 0.23, *P* < 0.001), but not with deprivation score (Spearman’s rank coefficient = 0.01, *P* = 0.396). There was significant evidence that the data for leukaemia, lymphoma and CNS tumours were over dispersed (likelihood ratio test for the scale parameter being equal to zero gave *P =* 0.013, *P =* 0.005 and *P* < 0.001 respectively). Negative binomial regression models were therefore fitted in favour of Poisson regression models. Incidence rate ratios (IRRs) obtained via negative binomial regression models for population mixing, deprivation scores and population density by diagnostic group are given in Table 2, and for population mixing and deprivation by diagnostic subgroup in Table 3. Model comparisons showed that for all diagnostic groups the best fitting model was obtained by including population mixing and deprivation in their continuous forms, except for leukaemia in which the inclusion of deprivation score did not improve the model fit (Table 4). Person-weighted population density did not improve the model fit for any diagnostic group.

**Table 2:** **Incidence rate ratios (IRR) and 95% confidence intervals (CI) for leukaemia, lymphoma and CNS tumours in teenagers and young adults, 1996–2005**

		Leukaemia			Lymphoma			CNS Tumours ^b^		
Model ^a^	Variables	IRR	95% CI	P-value	IRR	95% CI	P-value	IRR	95% CI	P-value
A	Population Mixing	**0.92**	**0.83-1.03**	**0.144**	0.94	0.87-1.01	0.086	0.81	0.74-0.89	<0.001
B	Population Mixing	0.93	0.83-1.04	0.222	**0.97**	**0.90-1.04**	**0.426**	**0.83**	**0.75-0.91**	**<0.001**
	Deprivation Score	0.99	0.98-1.00	0.114	**0.97**	**0.96-0.98**	**<0.001**	**0.97**	**0.96-0.99**	**<0.001**
C	Population Mixing	0.95	0.84-1.07	0.446	0.97	0.90-1.06	0.529	0.82	0.74-0.91	<0.001
	Deprivation Score	0.99	0.98-1.01	0.609	0.97	0.96-0.98	<0.001	0.97	0.95-0.99	0.001
	Population Density^c^	1.00	0.99-1.00	0.433	1.00	1.00-1.00	0.820	1.00	0.99-1.00	0.675
D	Population Mixing									
	1st Decile	0.93	0.73-1.19	0.572	0.97	0.83-1.14	0.689	1.35	1.13-1.61	0.001
	2-9^th^ Decile	1	-	-	1	-	-	1	-	-
	10^th^ Decile	0.9	0.77-1.05	0.168	0.98	0.89-1.09	0.718	0.93	0.81-1.06	0.267
	*Test for Trend*			*0.203*			*0.778*			*0.075*
	Deprivation Score	0.99	0.98-0.99	0.163	0.97	0.96-0.98	<0.001	0.97	0.96-0.98	<0.001

^a^Best fitting model is highlighted in bold for each diagnostic group, model fit statistics are given in Table 4.

^b^Analysis for main CNS tumour subgroups are shown in Table 3.

^c^Person-weighted population density.

**Table 3:** **Incidence rate ratios (IRR) and 95% confidence intervals (CI) for main subgroups of leukaemia, lymphoma and CNS tumours in teenagers and young adults, 1996-2005**

Diagnostic subgroup	Multivariable model variables					
	Population mixing			Deprivation score		
	IRR	95% CI	***P***	IRR	95% CI	***P***
**Leukaemia**						
Acute lymphoid leukaemia	0.96	0.82-1.13	0.658	0.99	0.97-1.01	0.149
Acute myeloid leukaemia	0.88	0.73-1.06	0.168	0.99	0.97-1.01	0.474
Chronic myeloid leukaemia	0.97	0.71-1.34	0.862	1.01	0.98-1.05	0.497
Other leukaemia	0.89	0.59-1.34	0.575	0.95	0.91-1.00	0.074
**Lymphoma**						
Non-Hodgkin lymphoma	1.02	0.90-1.16	0.710	0.98	0.97-1.00	0.038
Hodgkin lymphoma	0.94	0.86-1.03	0.204	0.96	0.95-0.97	<0.001
**CNS Tumours**						
Astrocytoma	0.83	0.71-0.98	0.025	0.98	0.96-1.00	0.069
Other Glioma	0.77	0.57-1.03	0.076	0.96	0.92-0.99	0.026
Ependymoma	0.97	0.65-1.44	0.863	0.96	0.91-1.01	0.092
Medulloblastoma	1.10	0.75-1.60	0.625	0.96	0.92-1.01	0.107
Other CNS tumours^a^	0.79	0.69-0.91	0.001	0.97	0.95-0.99	<0.001

^a^Includes groups 3.5 and 3.6. from Birch Classification Scheme [24].

**Table 4:** **Comparison of models shown in Table** 2

Models	Likelihood ratio test	AIC		Model choice
	***P***-value	Model 1	Model 2	
Leukaemia				
A and B	0.112	6900.5	6899.942	A
B and C	0.428	6899.9	6901.32	
B and D	0.503	6899.9	6901.33	
Lymphoma				
A and B	<0.001	11930	11887.95	B
B and C	0.819	11888	11889.90	
B and D	0.574	11888	11890.32	
CNS Tumours				
A and B	<0.001	8901.00	8882.79	B
B and C	0.676	8882.79	8884.61	
B and D	0.168	8882.79	8887.68	

For the best fitting models, population mixing did not significantly affect the incidence of leukaemia or lymphoma. However, increased levels of population mixing were associated with a significant decrease of 17% in incidence of CNS tumours for each unit increase in the Shannon index (IRR = 0.83, 95% CI = 0.75-0.91); this is equivalent to an overall incidence rate of 40.5 per million person-years for wards in the lowest centile of population mixing, compared to 26.8 per million person-years in the highest centile of population mixing. The effect of population mixing amongst CNS tumours was driven by astrocytoma (IRR = 0.83, 95% CI = 0.71-0.98) and ‘other CNS tumour’ subgroups (IRR = 0.79, 95% CI = 0.68-0.92).

Levels of deprivation were not associated with the incidence of leukaemia, however, there was a significant 3% decrease in incidence of lymphoma associated with more deprived areas (IRR = 0.97, 95% CI = 0.96-0.98), which was consistently observed for Non-Hodgkin’s and Hodgkin’s lymphoma (Table 3). Furthermore, a significant 3% decrease in incidence of CNS tumours was associated with increased deprivation (IRR = 0.97, 95% CI = 0.96-0.99). This effect was also observed within the ‘other glioma’ (IRR = 0.96, 95% CI = 0.92-1.00) and ‘other CNS tumour’ (IRR = 0.97, 95% CI = 0.0.95-0.99) subgroups (Table 3).

## Discussion

In the first major study investigating the association between population mixing and TYA cancer, we have shown that higher levels of mixing are significantly associated with decreased incidence of CNS tumours amongst TYAs in England. The incidence of CNS tumours in wards with the highest levels of population mixing was 1.5 times higher compared to the incidence of CNS tumours in wards with the lowest levels of population mixing. A similar magnitude of effect was observed amongst the astrocytoma and ‘other CNS tumour’ subgroups. Population mixing levels were not significantly associated with the incidence of ependymoma, medulloblastoma and other glioma subgroups, although this is not surprising given that these are much smaller subgroups (5%, 6% and 10% of total CNS tumours respectively). Population mixing and deprivation did not appear to affect incidence of leukaemia in TYAs, contrasting to evidence of a significant association between both covariates and the incidence of leukaemia in children [6, 11]. Similarly, in our previous work focusing on seasonality of birth and diagnosis, our results of TYAs with leukaemia contrasted to those for children with leukaemia [25]. These differences are likely to occur due to differences in the disease epidemiology of leukaemia between these two age groups, whereby ALL dominates childhood leukaemia in contrast to AML more commonly seen in TYAs and the ratio of T-cell to B-cell ALL is higher amongst TYAs compared to children [33–35]; thus suggesting that the aetiology of leukaemia amongst TYAs is very different to that of leukaemia amongst children.

Significantly decreased incidence rates for Hodgkin’s and non-Hodgkin’s lymphoma, CNS tumours overall, ‘other gliomas’ and ‘other CNS tumours’ were associated with more deprived areas. This is consistent with Alston [23] who showed that increased levels of deprivation were associated with decreased incidence of lymphoma and CNS tumours amongst 13–24 year olds in England diagnosed between 1979–2001.

Person-weighted population density did not have a significant effect on the incidence of any of the main cancer groups studied. This could be explained as population mixing was significantly correlated with population density. Future aetiological work should therefore focus on disentangling the confounding effects of population mixing and population density.

Higher levels of population mixing associated with a reduced risk of CNS tumours may have occurred through a protective effect for exposure to infection, since population mixing has been shown to be a reasonable proxy for infectious exposure [36]. One possible mechanism may involve immune system priming in early life by exposure to a wide range and high volume of infection, and may be important in terms of protection against later development of CNS tumours among TYAs.

Our interest in assessing whether there was any relationship between population mixing and the incidence of TYA cancer was motivated through increasing evidence that infections may play a role in the development of certain childhood cancers such as leukaemia [8, 37], lymphoma and CNS tumours [38], the latter specifically giving evidence of space-time clustering for astrocytoma in older children. Only one specific infectious agent, the polyomavirus, has been implicated in the disease aetiology for CNS tumours [39]. Besides associations with population mixing and deprivation, we have also shown that certain CNS tumours exhibit seasonality around the time of birth (with peaks in May and November for glioma) and diagnosis (peaks in December and June) [25].

### Strengths and limitations

This was the first national population-based study to evaluate the risk of TYA cancer in relation to population mixing, adjusting for the potentially confounding effects of deprivation and population density. As previously stated, use of population mixing has been shown to be a reliable proxy measure for infection in ecological study designs [36].

Our study design was an aggregation of individual counts of cancer occurring at ward level, thus the reported associations may be subject to the ecological fallacy [40]. Nevertheless, census wards are small, with a 15–24 year old population of around 900 people per ward on average. Furthermore, wards have been shown to have relatively homogeneous population sizes when compared to larger geographical areas such as district level [11], in our case the 15–24 year old population ranged in size from 126 to 4015 per ward for 98% of wards. In addition we were able to link all cases in the study to a census ward thus avoiding any selection bias. We chose to allocate levels of population mixing, deprivation and population density based on matching cases to the 1991 census. Although relative levels of population mixing do not change markedly from one census to the next (Dr Zhiqiang Feng, personal communication), this could have led to measurement error with cases born between 1971 and 1990 and diagnosed between 1996 and 2005, with the possibility that cases may have moved residence several times between birth and diagnosis. Nor were we able to distinguish whether time since exposure or age at exposure may have been of greater importance given the age range and period of diagnosis of subjects included in the analysis. The key timing window of exposure to levels of mixing in relation to the risk of cancer in TYAs is uncertain, particularly for CNS tumours which comprise a number of different benign tumours with long latency periods and potentially distinct aetiologies.

## Conclusion

In summary, there is an emerging epidemiological picture implicating an environmental aetiology for TYA cancers, especially for certain CNS tumours, and future work examining correlates of population mixing and deprivation would be worthwhile. This could be achieved by undertaking a more comprehensive and focused geographical analysis, for example by identifying specific areas or regions, which exhibit significantly elevated incidence for CNS tumours and correlating these to levels of circulating infection in the community over several decades. Individual residential and exposure histories documenting infectious episodes would be valuable to discern the association between infection and the risk of TYA cancers. This could be achieved by linking primary and secondary care data to national cancer registration data to evaluate consultations and admissions related to infection.

## References

[1] Kinlen L (1988). Evidence for an infective cause of childhood leukaemia: comparison of a Scottish new town with nuclear reprocessing sites in Britain. Lancet.

[2] Dickinson H, Hammal D, Bithell J, Parker L (2002). Population mixing and childhood leukaemia and non-Hodgkin's lymphoma in census wards in England and Wales, 1966–87. Br J Cancer.

[3] Law GR, Parslow RC, Roman E (2003). Childhood cancer and population mixing. Am J Epidemiol.

[4] Greaves M (1997). Aetiology of acute leukaemia. Lancet.

[5] Anderson RM, May R (1990). Immunisation and herd immunity. Lancet.

[6] Parslow RC, Law GR, Feltbower R, Kinsey SE, McKinney PA (2002). Population mixing, childhood leukaemia, CNS tumours and other childhood cancers in Yorkshire. Eur J Cancer.

[7] Basta NO, James PW, Craft AW, McNally RJQ (2010). Season of birth and diagnosis for childhood cancer in Northern England, 1968–2005. Paediatr Perinat Epidemiol.

[8] McNally RJQ, Eden TOB (2004). An infectious aetiology for childhood acute leukaemia: a review of the evidence. Br J Haematol.

[9] Simpson J, Smith A, Ansell P, Roman E (2007). Childhood leukaemia and infectious exposure: a report from the United Kingdom Childhood Cancer Study (UKCCS). Eur J Cancer.

[10] Chang JS, Tsai C-R, Tsai Y-W, Wiemels JL (2012). Medically diagnosed infections and risk of childhood leukaemia: a population-based case–control study. Int J Epidemiology.

[11] Stiller C, Kroll M, Boyle P, Feng Z (2008). Population mixing, socioeconomic status and incidence of childhood acute lymphoblastic leukaemia in England and Wales: analysis by census ward. Br J Cancer.

[12] Hjalgrim H, Engels E (2008). Infectious aetiology of Hodgkin and non-Hodgkin lymphomas: a review of the epidemiological evidence. J Intern Med.

[13] McNally RJQ, Stiller C, Vincent TJ, Murphy MFG (2014). Cross-space-time clustering of childhood cancer in Great Britain: Evidence for a common aetiology. Int J Cancer.

[14] McNally RJ, James PW, Picton SV, McKinney PA, van Laar M, Feltbower RG (2012). Space-time clustering of childhood central nervous system tumours in Yorkshire, UK. BMC Cancer.

[15] Ortega-García JA, López-Hernández FA, Fuster-Soler JL, Martínez-Lage JF (2011). Space–time clustering in childhood nervous system tumors in the Region of Murcia, Spain, 1998–2009. Childs Nerv Syst.

[16] Schmidt L, Grell K, Frederiksen K, Johansen C, Schmiegelow K, Schüz J (2009). Seasonality of birth in children with central nervous system tumours in Denmark, 1970–2003. Br J Cancer.

[17] McNally RJ, Alston RD, Eden TO, Kelsey AM, Birch JM (2004). Further clues concerning the aetiology of childhood central nervous system tumours. Eur J Cancer.

[18] Harding N, Birch J, Hepworth S, McKinney P (2009). Infectious exposure in the first year of life and risk of central nervous system tumors in children: analysis of day care, social contact, and overcrowding. Cancer Causes Control.

[19] McNally RJQ (2010). Are early infectious exposures involved in the etiology of childhood CNS tumors?. Expert Rev Neurother.

[20] Schmidt L, Kamper-Jørgensen M, Schmiegelow K, Johansen C, Lähteenmäki P, Träger C, Stokland T, Grell K, Gustafson G, Kogner P (2010). Infectious exposure in the first years of life and risk of central nervous system tumours in children: analysis of birth order, childcare attendance and seasonality of birth. Br J Cancer.

[21] Birch JM, Alston RD, Quinn M, Kelsey AM (2003). Incidence of malignant disease by morphological type, in young persons aged 12–24 years in England, 1979–1997. Eur J Cancer.

[22] Bleyer WA (2002). Cancer in older adolescents and young adults: epidemiology, diagnosis, treatment, survival, and importance of clinical trials. Med Pediatr Oncol.

[23] Alston RD, Rowan S, Eden TOB, Moran A, Birch JM (2007). Cancer incidence patterns by region and socioeconomic deprivation in teenagers and young adults in England. Br J Cancer.

[24] Birch JM, Alston RD, Kelsey AM, Quinn MJ, Babb P, McNally RJQ (2002). Classification and incidence of cancers in adolescents and young adults in England 1979–1997. Br J Cancer.

[25] van Laar M, Kinsey SE, Picton SV, Feltbower RG (2013). First description of seasonality of birth and diagnosis amongst teenagers and young adults aged 15–24 with cancer in England, 1996–2005. BMC Cancer.

[26] Rhodes C, Anderson R (1996). Persistence and dynamics in lattice models of epidemic spread. J Theor Biol.

[27] Miller GA (1955). Note on the bias of information estimates. Inf Theory Psychol.

[28] Office of Population Censuses and Surveys (1991). Census: special migration statistics (Great Britain). ESRC/JISC Census Programme.

[29] Dorling D, Atkins D, Office of Population Censuses & Surveys [OPCS] (1995). Population density, change and concentration in Great Britain 1971, 1981 and 1991. Studies on Medical & Population Subjects No. 58.

[30] Townsend P, Phillimore P, Beattie A (1988). Health and deprivation: inequality and the North.

[31] Akaike H, Petrov BN, Czàke F (1973). Information theory and an extension of the maximum likelihood principle. Second International Symposium on Information Theory.

[32] Feltbower RG, Manda SOM, Gilthorpe MS, Greaves MF, Parslow RC, Kinsey SE, Bodansky HJ, McKinney PA (2005). Detecting small-area similarities in the epidemiology of childhood acute lymphoblastic leukemia and diabetes mellitus, type 1: a Bayesian approach. Am J Epidemiol.

[33] McKinney P, Alexander F, Cartwright R, Scott C, Staines A (1993). Acute lymphoblastic leukaemia incidence in the UK by immunophenotype. Leukemia.

[34] Marks DI, Paietta EM, Moorman AV, Richards SM, Buck G, DeWald G, Ferrando A, Fielding AK, Goldstone AH, Ketterling RP (2009). T-cell acute lymphoblastic leukemia in adults: clinical features, immunophenotype, cytogenetics, and outcome from the large randomized prospective trial (UKALL XII/ECOG 2993). Blood.

[35] Greaves MF, Janossy G, Peto J, Kay H (1981). Immunologically defined subclasses of acute lymphoblastic leukaemia in children: their relationship to presentation features and prognosis. Br J Haematol.

[36] Taylor JC, Law GR, Boyle PJ, Feng Z, Gilthorpe MS, Parslow RC, Rudge G, Feltbower RG (2008). Does population mixing measure infectious exposure in children at the community level?. Eur J Epidemiol.

[37] Greaves M (2002). Science, medicine, and the future: childhood leukaemia. BMJ.

[38] McNally R, Cairns D, Eden O, Alexander F, Taylor G, Kelsey A, Birch J (2002). An infectious aetiology for childhood brain tumours? Evidence from space–time clustering and seasonality analyses. Br J Cancer.

[39] Love S, Louis DN, Ellison D (2008). Greenfield’s neuropathology.

[40] Piantadosi S, Byar DP, Green SB (1988). The ecological fallacy. Am J Epidemiol.

[CR41] The pre-publication history for this paper can be accessed here:http://www.biomedcentral.com/1471-2407/14/698/prepub

